# Development of a Conceptual Framework for Smart Hospitals: A Qualitative Study in Iran

**DOI:** 10.1002/hsr2.71339

**Published:** 2025-11-18

**Authors:** Roya Malekzadeh, Ghasem Abedi, Majid Sadeghi

**Affiliations:** ^1^ Department of Healthcare Services Management, Health Sciences Research Center, Faculty of Health Mazandaran University of Medical Sciences Sari Iran; ^2^ Department of Healthcare Services Management, Faculty of Health Mazandaran University of Medical Sciences Sari Iran

**Keywords:** hospital, intelligentization, model, smart hospital

## Abstract

**Background and Aims:**

The concept of a smart hospital refers to an advanced care model aimed at improving quality of care while using resources efficiently. This study aims to develop a comprehensive conceptual model that clearly defines the key dimensions of smart hospitals.

**Methods:**

This qualitative study was conducted in 2024. The participants included 22 health system managers with a minimum of 5 years of managerial experience, alongside policymakers and scientific experts familiar with the concept of smart hospitals in Iran. Data were collected through targeted semi‐structured interviews, followed by snowball sampling until data saturation was reached. The interview questions focused on the concepts and dimensions of smart hospitals. Subsequently, the interviews were transcribed, meaning units were identified, coded, and classified based on similarities and patterns. Themes were then extracted and analyzed using content analysis.

**Results:**

The analysis identified 5 main themes, 21 subthemes, and 94 items. These themes included: Management and Leadership (control, human resource, financial resource, planning, and management dashboard), Care and Treatment (inpatient services, outpatient services, pharmaceutical services, and health promotion), Information and Communication Systems (health files, communication infrastructure, information infrastructure, and network management), Medical Equipment (therapeutic equipment, diagnostic equipment, detection equipment, and rehabilitation equipment), and Structures and Facilities (energy management, risk management, waste management, and smart facilities).

**Conclusion:**

The smart hospital concept is complex, requiring multidisciplinary collaboration and adherence to health standards. Success depends on optimizing resources, strengthening policies and planning, enhancing information systems, upgrading facilities, and prioritizing smart care processes and medical equipment.

## Summary

- The concept of a smart hospital represents a comprehensive care model aimed at improving care quality and ensuring sustainable resource utilization.
- Key objectives include reducing medical errors, enhancing patient safety, achieving cost efficiency, automating processes, and adapting to emerging technologies.
- To achieve this vision, it is essential to optimize existing capacities, strengthen policy and planning components, and organize resources effectively and efficiently.

## 1 Introduction

In today's world, smart technologies are widely used across various sectors, including finance, commerce, healthcare, tourism, and industry [1]. With rapid advances in information and communication technologies, leveraging smart tools has become critical for competitive strategies [2]. Healthcare is no exception, as transformative technologies significantly improve longevity and quality of life. The emergence of smart hospitals and advanced medical equipment is revolutionizing patient care [3]. However, as healthcare systems grow more complex and data‐driven, traditional data management methods are inadequate, increasing the risk of poor outcomes. Intelligent solutions are therefore essential [4].

In recent years, medicine has shifted from disease treatment to a prevention‐oriented, consumer‐centered approach. Hospitals now integrate AI, robotics, and fourth industrial revolution technologies to improve diagnosis and treatment quality [5]. A smart hospital is defined as a facility using optimized, automated processes in an interconnected ICT environment to enhance patient care and enable innovative diagnostic and treatment methods [6]. Its core goal is to deliver optimal services with minimal errors and at lower costs by leveraging advanced ICT [7].

The concept of a smart hospital aligns with a comprehensive care model aimed at improving care quality and ensuring sustainable resource use [8]. Its objectives include advanced patient care, remote medical services, enhanced diagnostics, and rapid response to patient needs [9]. Smart technologies can address healthcare limitations by closing supply gaps and increasing service accessibility through ICT adoption. According to WHO (2018), digital health solutions could transform health standards, treatment outcomes, and access to services promoting health and well‐being [10].

The concept of a smart hospital is complex, requiring multidisciplinary collaboration and compliance with numerous health standards. Although still unrealized, rapid technological advances promise a strong future for healthcare [11]. While the term “smart” has been compared to the SMART framework, it has not been systematically applied in hospitals [5]. Despite the benefits of AI and automation, adoption in healthcare remains slow [12]. Given the accelerating maturity and adoption of innovative technologies, defining the dimensions and requirements of smart hospitals is now essential.

In the past, low energy costs and limited patient–provider communication needs reduced the urgency for smart hospitals. Today, with rapid technological progress and increasingly complex healthcare delivery, their development has become indispensable. The demand for continuous patient–physician interaction, real‐time monitoring of patients and staff, error reduction, enhanced patient safety, automation, and economic efficiency—together with technologies like IoT—highlights this necessity [13]. Yet, most studies have narrowly focused on individual technologies, hospital information systems, or building design [14]. The concept of a smart hospital extends beyond digital tools and requires a holistic, managerial, and systemic approach [15]. Critically, no comprehensive conceptual or managerial model currently exists, representing a major research gap. Without such an integrated framework—encompassing organizational, strategic, and managerial dimensions—efforts at hospital intelligentization risk remaining fragmented, overly technology‐driven, and misaligned with the broader objectives of health system transformation and sustainable service improvement. Addressing this gap is therefore essential to guide effective implementation and ensure that smart hospital initiatives achieve their full potential. Accordingly, this study seeks to develop a localized conceptual model for smart hospitals in Iran's health system.

The model aims to provide a robust theoretical and practical framework to guide policymakers, planners, and senior managers in improving health service quality, reducing costs, and advancing hospital digital transformation.

## 2 Material and Methods

This qualitative study, conducted in 2024, used content analysis to develop a conceptual model for smart hospitals.

Participants included staff managers from the Ministry of Health and medical universities nationwide, expert policymakers in smart hospitals, and scientific experts or doctoral graduates in healthcare management, health policy, or emerging technologies such as artificial intelligence (AI).

Inclusion criteria required at least 5 years of management experience and expertise relevant to smart hospitals.

Participants were selected through purposive sampling and snowball techniques to ensure broad representation of key stakeholders. Interviews continued until data saturation was reached, ensuring no new information emerged.

In‐depth, semi‐structured interviews were conducted until data saturation to gather expert insights.

An interview guide was developed based on a comprehensive literature review (including Kwon and colleagues, Tian and colleagues, and Yadegari and Asosheh) and refined through expert consultations [5, 12, 16]. The guide's validity and relevance were confirmed and improved via expert feedback, ensuring conceptually and contextually appropriate questions.

Interview questions explored participants' understanding of smart hospital concepts and dimensions.

Participants were encouraged to define the term “smart hospital,” provide examples, and share experiences (including during COVID‐19). Sample questions included:

- What comes to mind when you hear the term “smart hospital”?
- Can you explain the concept and dimensions of a smart hospital?
- Please share examples of smart hospital practices you have observed.

Follow‐up and exploratory questions were used as needed to clarify responses and deepen understanding.

Interviews were scheduled in advance and coordinated by phone or in person.

Participants were informed about the study's purpose, assured of confidentiality, and told they could withdraw at any time. Written informed consent was obtained from all participants.

To ensure accuracy and privacy, interviews were conducted in quiet locations away from workplace distractions. Each interview lasted an average of 55 ± 10 min.

The qualitative approach of Granheim and Lundman was employed for content analysis. Interviews were transcribed and repeatedly reviewed for an overall understanding. Sentences, paragraphs, or words were treated as meaning units, summarized, and coded to capture underlying meanings. Codes were then compared, grouped into broader categories, and finally synthesized into overarching themes relevant to the study.

To ensure the study's validity and rigor, Guba and Lincoln's criteria were applied. Credibility was enhanced through prolonged engagement with participants, careful data collection, and validation of findings. To ensure intercoder reliability, two independent researchers coded a subset of the interviews and discussed discrepancies until consensus was reached. Additionally, member checking was conducted by sharing the synthesized findings with several participants to validate accuracy and relevance. Dependability and confirmability were strengthened through faculty and expert feedback, while transferability was supported by providing a detailed research report with direct participant quotations.

The Delphi technique was used to validate and finalize the components and dimensions of the conceptual model derived from the qualitative phase. An expert panel of 12 specialists in hospital management and research team members was formed, which was considered appropriate given the group's homogeneity. A questionnaire based on the qualitative findings assessed each component's importance and feasibility on a 9‐point Likert scale (1 = *completely inappropriate*, 9 = *completely appropriate*). Scores 1–3 were rejected, 4–6 required further review, and 7–9 were accepted. Components scoring below 4 were removed; those scoring 7 or higher were accepted; and those between 4 and 7 advanced to subsequent Delphi rounds. Expert participation was maintained above 70% in each round, and consensus was assumed when score changes between rounds were < 15%.

## 3 Results

In the qualitative phase, 22 managers from the Ministry of Health and medical universities across the country participated. Of these, 77% were male and 23% female; 91% held doctoral degrees and 9% held master's degrees. Detailed demographic characteristics are presented in Table 1.

**Table 1:** Demographic characteristics of the participants.

Variable		*N* (%)
Sex	Male	(77) 17
	Female	(23) 5
Education	Ph.D	(91) 20
	MSc	(9) 2
	BSc	(0) 0
Age	Above 50	(81.8) 18
	40–50	(13.7) 3
	30–40	(4.5) 1
	20–30	(0) 0
Work history	Above 15	(54.5) 12
	20–25	(27.3) 6
	15–20	(13.4) 3
	10–15	(4.5) 1
	Under 10	(0) 0

The analysis generated 320 initial codes, which were subsequently organized into 5 main themes: management and leadership, care and treatment, health communication and information, medical equipment, and structures and facilities. These themes encompassed 21 subthemes and 94 specific items (Table 2).

**Table 2:** Extraction of the main and sub‐themes of the smart hospital.

Main theme	Sub‐theme	Items
Management and leadership	Control	Permission and access control
		Supervising the warehouse of materials and supplies
		Customer traffic control
		Supervision of medicinal items
	Human resource	Manpower planning
		Track the exact location of employees
		Smart check‐in and check‐out system
		Capacity building and Smart training of employees
		Development of office automation
	Financial resource	Allocation of budget for information technology and digital transformation
		technology Investment in AI‐ and IoT‐based infrastructures
		Operational budgeting for system maintenance
		Budgeting for digital training
		Program for resource mobilization through public‐private partnerships
	Planning	Policy formulation
		Strategic and operational planning by AI
		Measuring performance indicators
		Analysis of the results
		Artificial intelligence processing decision system
	Management dashboard	Intelligent statistics of information
		Stakeholder satisfaction
		Data analysis and analysis
		Report feedback
Care and treatment	Inpatient services	Nursing care
		Disease diagnosis
		Analysis and prediction of disease
		Patient safety
	Outpatient services	Virtual clinic
		Remote care
		Triage patients
		Online appointment
	Pharmaceutical services	Drug validation
		Robotic pharmacy
		Drug incompatibility control
		Drug prescribing decision support system
	Preventive services & health promotion	Public health monitoring using wearable devices
		AI algorithms for risk factor identification
		Automated alerts for preventive actions
		App‐based health education
		Educating patients and companions
		Remote consultation
Health communication and information	Health file	Electronic health record
		Electronic prescribing
		Sharing patient information
		Archive system and image exchange
	Communication infrastructure	artificial intelligence
		Development of virtual communication
		Health tourism virtual tour
		Traffic engineering
	Information infrastructure	Design of a centralized and secure data architecture
		Data storage
		Automation
		Services based on the Internet of Things
		Big data analysis
	Network management	Calibration of intelligent systems
		Security of intelligent systems
		Integration of information systems
		Development of medical knowledge and research
Medical equipment	Therapeutic equipment	Robot doctor friend
		Nurse assistant robot
		Smart bed and accessories
		Smart medical devices
	Diagnostic equipment	Smart sensors
		Smart imaging equipment
		Implants
		Laboratory equipment
	Detection and monitoring equipment	Intelligent patient identification equipment
		Smart patient monitoring equipment
		Intelligent infection detection system
		Intelligent detection of medical errors
	Rehabilitation equipment	Rehabilitation robot
		Transportation robot
		Intelligent artificial limbs
		Smart processor
Structures and facilities	Energy management	Smart lighting management
		Smart heating and cooling systems
		Smart irrigation and water supply systems
		Renewable energy generation
		Smart ventilation
	Risk management	Intelligent warning systems
		Fire extinguishing system
		Central paging
		UPS system
		Smart structural health monitoring
	Waste management	Smart waste collection
		Smart waste disposal
		Smart wastewater treatment
		Smart greywater management
	Smart facilities	Notice board and smart guide
		Smart amenities
		Smart parking system
		Environmental sustainability (Green hospital)
		Guide robot
		Smart architecture

### 3.1 Theme 1: Management and Leadership

Participants highlighted the pivotal role of management and leadership, noting that “hospitals are complex social organizations with dual responsibilities and numerous challenges. They emphasized that effective resource management is essential to address these complexities” (P3). Five subthemes were identified: control, human resources, financial resources, planning, and management dashboards.

#### 3.1.1 Control

Almost all participants stressed the importance of oversight in hospital management for effective performance measurement. One participant stated, “Every smart hospital must be equipped with systems for monitoring both employees and visitors” (P2). Another emphasized, “Given the smart capabilities of the hospital and the use of internet and communication systems for information exchange, access to information needs to be organized and regulated” (P6). Additionally, participants noted that “monitoring inventory and comparing it with expected levels and previous years' stock can improve productivity and support better planning” (P2).

#### 3.1.2 Human Resource

Participants emphasized the important role of AI in automating human resource (HR) tasks. One participant noted, “AI can automate salary and benefits management and is especially effective in speeding up contract and job description creation” (P7). Another explained, “AI automates repetitive tasks such as attendance and leave management, reducing paperwork” (P8). Additionally, “AI is used for workforce planning by analyzing skills, industry trends, and growth plans to predict hiring needs” (P3). Participants also stressed the value of investing in technology‐driven training programs that “enhance skills, foster innovation, and ensure workforce adaptability for future challenges” (P11).

#### 3.1.3 Financial Resource

Participants emphasized the critical challenge of budget allocation for digital transformation. One participant stated, “A major challenge is allocating enough budget for IT. Without sufficient investment in AI and IoT infrastructure, system efficiency and intelligence cannot be achieved” (P4). Another noted, “System maintenance and support are integral to our operational budget. Poor planning of repair and update costs negatively impacts technology performance” (P11). Additionally, “Digital training for employees and resource mobilization through public–private partnerships are vital for successful technology implementation and financial efficiency” (P9).

#### 3.1.4 Planning

Participants highlighted planning as a core managerial responsibility, emphasizing that hospital managers should lead strategic planning and define performance indicators. One participant noted, “AI can greatly assist in this process” (P1 and P11). Another added, “Decision Support Systems (DSS) analyze large data sets to suggest the best options for the organization” (P7).

#### 3.1.5 Management Dashboard

Participants emphasized that “having a smart management dashboard featuring key performance indicators and client satisfaction reports is essential for effective decision‐making” (P2). Another stated, “Such tools enable quick responses to environmental changes, identify market opportunities, promote a learning organization culture, and maximize productivity” (P17).

### 3.2 Theme 2: Care and Treatment

Most participants believed that a smart hospital is meaningless without smart care and treatment, making this one of the core aspects of a smart hospital. This theme included four subthemes: inpatient services, outpatient services, pharmaceutical services, and preventive services and health promotion.

#### 3.2.1 Inpatient Services

Participants emphasized the role of medical devices and AI in inpatient care. One noted, “Medical devices like heart monitors track vital signs, and AI collects this data to diagnose complex conditions such as sepsis” (P22). Another stated, “Deep neural networks can identify causes of diabetic retinopathy and macular edema, enabling earlier diagnosis often challenging for ophthalmologists” (P11). Additionally, “Holographic imaging systems, similar to augmented reality, provide 3D visualization of organs like the heart without special glasses” (P19).

#### 3.2.2 Outpatient Services

Participants highlighted the role of AI in improving outpatient care. One stated, “AI‐powered virtual assistants can provide patients with 24/7 support based on their medical history and needs” (P10). Another emphasized, “Automating patient admission through self‐service kiosks reduces waiting times and allows staff to focus more on patient needs” (P3). Additionally, “Online appointment systems and IVR help organize hospital visits and prevent overcrowding” (P12 and P19).

#### 3.2.3 Pharmaceutical Services

Participants stressed the importance of tracking medications to prevent shortages. One noted, “RFID technology minimizes inventory management time and ensures accurate drug quantities” (P14). Another added, “Some hospitals and manufacturers use RFID tags to authenticate medications and combat counterfeiting” (P15). Furthermore, “By scanning prescription barcodes, pharmacist assistant robots can package medications and assist in storage and distribution” (P15). Additionally, “AI algorithms review prescriptions for potential drug interactions and can automatically notify patients about medication recommendations” (P17).

#### 3.2.4 Preventive Services and Health Promotion

Participants highlighted the use of *wearable devices for continuous health monitoring*. One stated, “Real‐time data combined with AI helps quickly identify risk factors and implement preventive measures” (P22). Another emphasized, “Automated alerts for preventive actions significantly improve outcomes and reduce the burden on healthcare facilities” (P8). Additionally, “App‐based health education programs and remote consultations empower patients and caregivers to manage health effectively at home” (P20).

### 3.3 Theme 3: Health Communication and Information

Most participants agreed that establishing and developing communication and information infrastructures is a key strategy for successful smart hospital implementation. This theme comprises four subthemes: “health file, communication infrastructure, information infrastructure, and network management.”

#### 3.3.1 Health File

Participants highlighted the importance of electronic health records in smart hospitals. One said, “Electronic files give doctors access to all patient information; they are durable and streamline tasks, saving time” (P13). Another noted, “In smart hospitals, files and prescriptions are electronic, enabling information sharing among doctors and nurses as needed” (P18). Additionally, “Systems like PACS are used for imaging storage and exchanging related data” (P16).

#### 3.3.2 Communication Infrastructure

Participants highlighted the importance of modern communication tools in smart hospitals. One stated, “Smartphones provide features like internet access and various applications” (P13). Another emphasized, “A smart hospital should offer online appointment scheduling and optimize these systems” (P18). Additionally, “Augmented and virtual reality can be used to promote health tourism and inform patients about hospital conditions” (P16). Moreover, “Optimizing data traffic flow and allocating communication resources are essential in a smart hospital” (P7).

#### 3.3.3 Information Infrastructure

Participants stressed the need for robust data storage in hospital data centers. One stated, “Given the large data volume in smart hospitals, storing it in the hospital′s data center is essential” (P17). Another noted, “IoT and connected devices enhance system performance and communication” (P21). Additionally, “Technologies like IoT and machine learning are expanding smart tools that assist doctors” (P13). Moreover, “Analyzing patient data uncovers hidden patterns for diagnosis and, combined with historical data, predicts medication needs, hospital stay duration, and postdischarge care” (P18).

#### 3.3.4 Network Management

Most participants emphasized the critical role of access control and security systems in safeguarding patients, staff, and hospital assets. One noted, “Intelligent devices like entry/exit control systems, alarms, surveillance cameras, X‐ray detectors, drug packaging, and lab tests improve tracking, reduce security risks, and enhance patient care reliability” (P16). Another stated, “Sensors monitor respiratory devices, pumps, and medical equipment, automatically alerting staff of malfunctions” (P3). Additionally, “Security measures such as encryption ensure data protection against unauthorized access” (P2).

### 3.4 Theme 4: Medical Equipment

Participants emphasized that advancements in technology have made smart hospital equipment a key factor in improving healthcare quality. This theme includes four subthemes: “therapeutic, diagnostic, detection, and rehabilitation equipment.”

#### 3.4.1 Therapeutic Equipment

Participants highlighted the vital role of advanced medical equipment in smart hospitals, including surgical devices and robotics. One noted, “Robots assist doctors by performing complex tasks with precision and flexibility beyond human capabilities” (P14). Another stated, “Nurse robots manage medication distribution and medical services” (P4). Additionally, “Robotics relieve staff from labor‐intensive tasks, offering safer and more cost‐effective treatments” (P11). Smart devices like infusion pumps and intelligent respiratory aids were also deemed indispensable for patient care (P21).

#### 3.4.2 Diagnostic Equipment

Participants highlighted the crucial role of AI in image interpretation and patient triage. One explained, “AI can efficiently review patient scans, identifying suspicious cases such as COVID‐19, tuberculosis, or cancer, allowing radiologists to focus on positive findings and expedite treatment” (P8). Additionally, “Telehealth technologies enable medical sensors to transmit vital data like blood sugar and blood pressure to healthcare providers, aiding diagnosis and medication decisions” (P12).

#### 3.4.3 Detection and Monitoring Equipment

Participants underscored the value of RFID and IoT technologies in enhancing hospital monitoring. One noted, “RFID can track employee locations, restrict access to sensitive areas, and prevent theft of medications and equipment” (P16). Another added, “RFID enables accurate patient identification and location tracking, improving monitoring and reducing errors” (P22). Additionally, “IoT‐based systems allow intelligent hand hygiene monitoring, recording compliance in real time to control disease and hospital‐acquired infections” (P18).

#### 3.4.4 Rehabilitation Equipment

Participants emphasized that smart rehabilitation devices make challenging therapies safer and more effective. One noted, “These devices accelerate treatment, make it more targeted, and ease the process for patients, families, and care teams” (P9). For instance, “The Myomo‐equipped wristband is a breakthrough for individuals with hand or arm paralysis, enabling movement in affected areas” (P10). Additionally, “Speech disorders can be intelligently diagnosed using audio signal processing systems” (P12).

### 3.5 Theme 5: Structures and Facilities

Participants identified hospital structures and facilities as a critical component of the smart hospital concept, comprising four subthemes: “energy management, risk management, waste management, and smart facilities.”

#### 3.5.1 Energy Management

Participants emphasized that smart hospitals aim to optimize energy resource management through smart devices and systems (P5). One explained, “Lighting adjusts automatically based on ambient brightness, and building temperatures are intelligently controlled to minimize energy use” (P16). Another noted, “Sensors and algorithms detect occupancy and adjust water and gas supply accordingly” (P20). Participants also highlighted the role of intelligent irrigation controllers that, “unlike traditional systems, measure weather, soil moisture, and plant needs to adjust irrigation in real time” (P11).

#### 3.5.2 Risk Management

Participants highlighted the importance of using smart technologies to manage potential risks. One explained, “Advanced sensors can detect smoke and fire and automatically extinguish the fire” (P7). Others noted, “Smart hospitals must have intelligent UPS systems for power outages and systems to manage medical gas shortages” (P20). Additionally, “An intelligent central paging system enables urgent messages and critical announcements to reach staff and patients quickly and without delay” (P13).

#### 3.5.3 Waste Management

Participants emphasized that smart technologies streamline hospital waste management. One explained, “A garbage‐collecting robot can intelligently gather waste and transport it to the appropriate location” (P5). Another noted, “Sensors installed in trash bins allow real‐time monitoring of their fill levels” (P19). In addition, “In smart wastewater treatment, sensors measure quantitative and qualitative indicators, and the central processor adjusts the treatment process based on this data” (P6). Finally, “AI can analyze purification data to identify inefficiencies and provide solutions for optimization” (P11).

#### 3.5.4 Smart Facilities

Participants highlighted the importance of integrating smart architecture and comfort features into hospital facilities. One noted, “A smart hospital could utilize guiding robots to help navigate and display routes” (P17). Another stated, “Elements like walls, windows, and doors should be equipped with smart technology” (P21). Additionally, “Smart hospitals provide comfort facilities for patients and companions, such as smart beds, mattresses, and tablets offering essential treatment information” (P5).

### 3.6 Integrated Conceptual Model

To provide a clearer conceptual and visual representation of the findings, an integrated conceptual model was developed from the extracted main and subthemes. This model demonstrates how the five main themes—management and leadership, care and treatment, health communication and information, medical equipment, and structures and facilities—interact and collectively advance the concept of smart hospitals. The 21 subthemes are organized under their respective main themes, reflecting the hierarchical and multidimensional nature of the framework.

**Figure 1:**
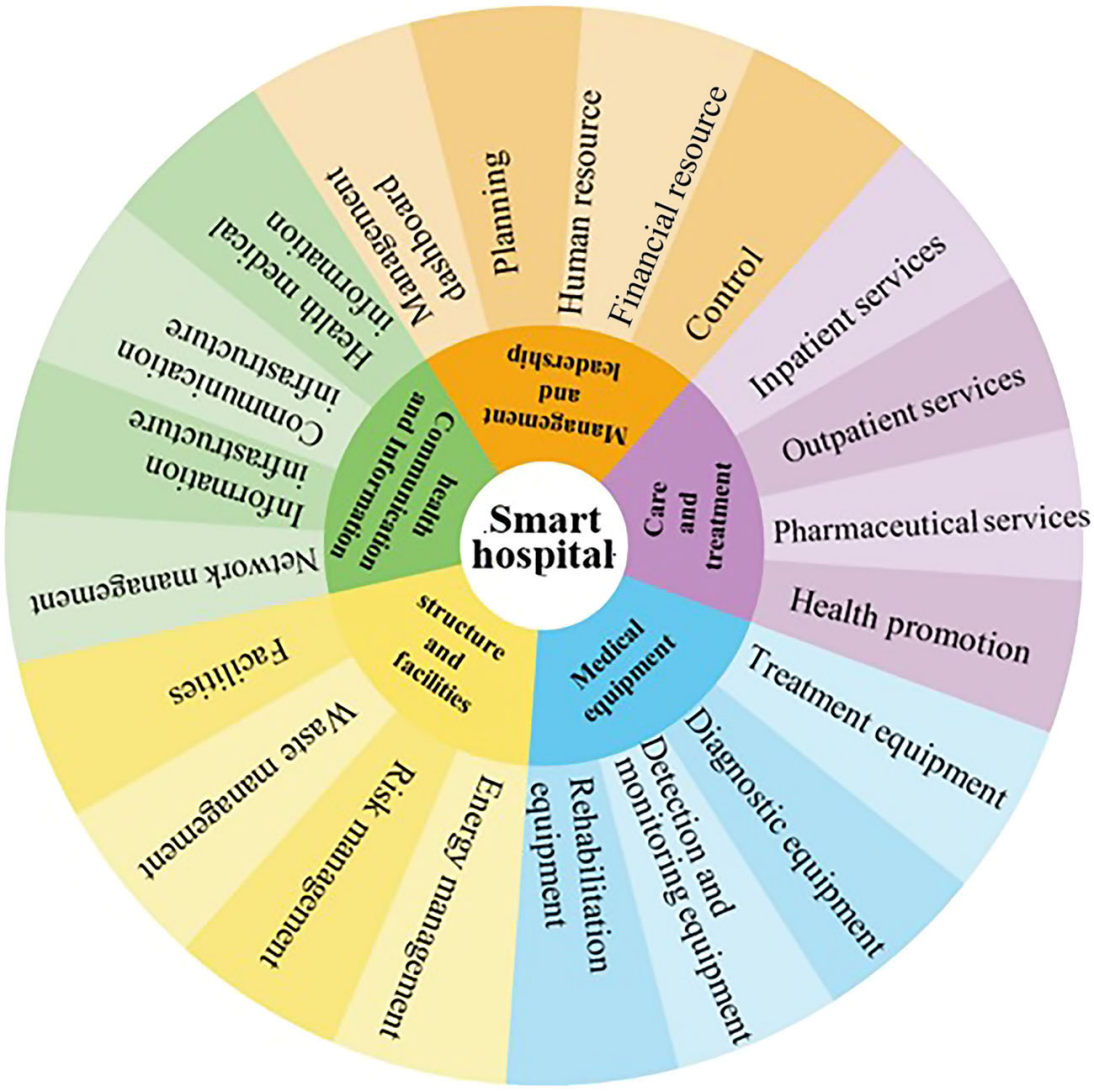
Conceptual model of the smart hospital.

The model was subsequently validated by experts through a specialized panel, ensuring its relevance and applicability within the healthcare context. Presenting this model (Figure 1) not only deepens the understanding of the qualitative findings but also clarifies the relationships among components. The framework can serve as a conceptual roadmap for policymakers, managers, and researchers, supporting strategic planning, action prioritization, and the design of evidence‐based interventions for smart hospital development and implementation.

## 4 Discussion

This study identified five main components of a smart hospital: Management and Leadership, Care and Treatment, Health Communication and Information, Medical Equipment, and Structures and Facilities. Previous research has categorized smart hospital dimensions differently. For example, Kwon and colleagues divided smart hospital services into eight areas, including location tracking, high‐speed networks, IoT, mobile health, AI‐driven services, robotics, augmented reality, and telehealth [5]. Yadegari and Asosheh proposed a service model based on IoT comprising infrastructure, core, and value‐added services [16]. Mirsaeidi Farahani and colleagues outlined seven themes focused on deployment planning, resource provision, organizational arrangements, and resistance management for smart service implementation in Iran [17]. While some findings align, differences arise due to varying contexts, timings, and methodologies. Unlike prior studies, this study emphasizes a comprehensive, integrated management perspective on hospital intelligentization, an area less addressed before.

This study identifies management and leadership as encompassing five subthemes: control, human resource, financial resources, planning, and management dashboard. GhaleSokhte et al. (2023) also emphasized management as crucial for smart hospital transformation, highlighting subthemes like hospital physical space, senior management support, energy management, control and monitoring, and change management [15]. Although some subthemes, such as physical space and energy management, are categorized differently here, Yadegari and Asosheh similarly stressed human resource management [16]. Due to the extensive clinical data, the management dashboard is critical for data interpretation and supporting decision‐making [18, 19]. Control and monitoring remain fundamental; Mirsaeidi Farahani and colleagues identified monitoring as a key subtheme and proposed intelligent individual monitoring aligned with this study for automation and information management [17].

The present study identifies treatment and care as comprising four subthemes: inpatient services, outpatient services, pharmaceutical services, and health promotion. Kwon and colleagues emphasized augmented reality's role in education and health promotion [5]. Holland and colleagues noted robotics applications in sterilization, disinfection, disease monitoring, and remote healthcare, aligning with this study [20]. Abdulkareem and colleagues highlighted machine learning and IoT use as clinical DSS to enhance diagnostic accuracy [21]. De Pretis and colleagues explored personalized pharmaceutical care via tailored brochures on drug risks and side effects [22]. Buddha and colleagues underscored AI and emerging technologies in disease detection, prevention, monitoring, and early treatment through patient data analysis [23]. Overall, smart care leverages AI to support evidence‐based decisions and improve patient outcomes.

Based on this study's findings, health information and communication include four subthemes: health file, communication infrastructure, information infrastructure, and network management. Effective data management involves accessing, integrating, controlling, and supervising data flow [24]. Kwon and colleagues identified smart hospital pillars as location identification, high‐speed networks, IoT infrastructure, and AI [5], though thematic classifications differ here. Abbas and colleagues proposed a blockchain‐based secure framework for patient data exchange [25]. Similarly, Yadegari and Asosheh emphasized communication, information, management, security, and privacy infrastructures as fundamental for smart hospital services [16]. Therefore, management must develop robust platforms and allocate resources to optimize these technologies for improved patient care [26].

This study classifies medical equipment into four subthemes: treatment, diagnostic, detection, and rehabilitation equipment. Advances in medical technology have raised demand for high‐quality, safe devices, requiring vigilant monitoring to prevent counterfeits and reduce errors [27]. Robotics are applied in surgery, rehabilitation, nursing, and logistics [5, 28]. Miniaturized sensors enable precise vital sign monitoring via body attachment or implantation [5]. Haque and colleagues developed an intelligent system improving hand hygiene to reduce hospital infections [29]. Rehabilitation robotics, such as the RIBA nursing robot aiding patient transfers, have advanced [30]. Smart devices like smart beds, infusion pumps, and RFID‐based patient ID wristbands are highlighted [31]. Overall, smart medical equipment in the Internet of Medical Things (IoMT) is essential for prompt emergency detection and clinical decision support [24].

This study identifies four subthemes within structures and facilities: energy management, risk management, waste management, and general facilities. Waste management is well‐studied [32], with blockchain‐based classification emerging as a promising solution [33, 34]. IoT‐enabled intelligent waste monitoring has been assessed for optimized disposal [35, 36]. Population growth and epidemics complicate medical waste disposal, where improper segregation damages the environment [37]. Hence, effective waste management and smart equipment integration are essential. Furthermore, IoT, AI, and digital technologies improve water supply management and address leakage issues [38, 39]. Smart building management encompasses energy, lighting, fire safety, water, heating, cooling, and air conditioning systems [40].

This study has limitations inherent to its qualitative design, as findings are context‐specific and may not fully generalize to other health systems with different structures and technological capabilities. However, maximum variation purposive sampling was employed to capture diverse perspectives across institutions and levels, enhancing data richness and transferability. Additionally, dual coding, member checking, and ongoing comparison with existing literature strengthened the credibility of the results.

## 5 Conclusion

Based on participants' experiences, 5 main themes and 21 subthemes emerged: management and leadership, care and treatment, health communication and information, medical equipment, and structures and facilities. The smart hospital concept is complex and multifaceted, requiring multidisciplinary collaboration and strict adherence to health standards. Therefore, it is recommended to optimize existing resources, strengthen policy and planning, efficiently organize resources, develop robust information and communication infrastructures, enhance facilities, and prioritize the intelligent integration of care processes and medical equipment.

## Author Contributions

**Roya Malekzadeh:** conceptualization, investigation, writing – original draft, methodology, validation, visualization, writing – review and editing, formal analysis, project administration, data curation, supervision, resources. **Ghasem Abedi:** conceptualization, investigation, data curation, writing – review and editing. **Majid Sadeghi:** investigation, writing – original draft, validation, visualization, writing – review and editing, software, formal analysis, data curation, resources. All authors have read and approved the final version of the manuscript.

## Ethics Statement

The procedures were approved by the Medical Ethics Committee of Mazandaran University of Medical Sciences (IR.MAZUMS.REC.1403.111).

## Consent

Written informed consent was obtained from all participants. Explicit consent for submission was secured from all coauthors and from the responsible authorities at the affiliated institution prior to submission. The purpose of this study was completely explained to the participants, and they were assured that their information would be kept confidential by the researcher. Informed consent from the participants was acquired as they agreed to participate in the study.

## Conflicts of Interest

The authors declare no conflicts of interest.

## Transparency Statement

The Roya Malekzadeh affirms that this manuscript is an honest, accurate, and transparent account of the study being reported and that no important aspects of the study have been omitted.

## Data Availability

All data relevant to the study are included in the article. Corresponding authors R.M. and M.S. had full access to all of the data in this study and take complete responsibility for the integrity of the data and the accuracy of the data analysis.
